# The Influence of Different Amaranth Leaf Meal Inclusion Levels on Performance, Blood Profiles, and Gut Organ Characteristics of Ross 308 Broiler Chickens

**DOI:** 10.3389/fvets.2022.869149

**Published:** 2022-05-04

**Authors:** Tlou Grace Manyelo, Nthabiseng Amenda Sebola, Jones Wilfred Ng'ambi, William Weeks, Monnye Mabelebele

**Affiliations:** ^1^Department of Agriculture and Animal Health, College of Agriculture and Environmental Sciences, University of South Africa, Florida, South Africa; ^2^Department of Agricultural Economics and Animal Production, University of Limpopo, Polokwane, South Africa; ^3^Agricultural Research Services, Department of Agriculture and Rural Development, Potchefstroom, South Africa

**Keywords:** amaranth, nutrient digestibility, performance, hematology, chickens

## Abstract

This study aimed to determine the effect of varying inclusion levels of amaranth leaf meal on the performance, blood profiles, and gut organ characteristics of Ross 308 broiler chickens. A total of 200, day-old, Ross 308 broiler chicks were randomly allocated to five dietary treatments in a complete randomized design, with each group having four replicates with ten chicks. Amaranth leaf meal (ALM) inclusion levels used in this study were 0, 5, 10, 15, and 20%. Body weight and feed intake were measured weekly to calculate the feed conversion ratio. Gut organ weights, lengths, organ pH, and blood profiles were measured and the general linear model of statistical analysis software was used to analyze collected data. ALM had no effect (*p* > 0.05) on feed intake, body weight, or the feed conversion ratio of Ross 308 broiler chickens between 1–21 and 22–42 days, respectively. Furthermore, ALM inclusion levels had no effect (*p* > 0.05) on dry matter (DM) or gross energy (GE) digestibility of Ross 308 broiler chickens. Ross 308 broiler chickens, which were fed with 5% ALM inclusion levels, had higher (*p* < 0.05) white blood cells, lymphocytes, and eosinophils than those fed with diets containing 0, 10, 15, and 20%. Chickens that were fed with 0 and 20% ALM inclusion levels had higher (*p* < 0.05) heterophils than those fed with diets containing 5, 10, and 15%. Chickens that were fed with 15% ALM inclusion levels had higher (*p* < 0.05) monocytes, eosinophils, and basophils than those fed with diets containing 0, 10, and 20%. Apart from Ile, ALM inclusion of 5 and 10% had higher (*p* < 0.05) essential and nonessential amino acid digestibility in Ross 308 broiler chickens. ALM inclusion levels had no effect (*p* > 0.05) on gut organ lengths or weights of Ross 308 broiler chickens aged 21 and 42 days. In conclusion, 5, 10, and 15% ALM inclusion levels can be included in broiler chicken diets as they showed favor in most of the affected parameters.

## Introduction

In South Africa and worldwide, the poultry sector contributes immensely to the global food security status of consumers (1). According to the Food and Agriculture Organization (FAO) (2), the demand for poultry feeds is increasing due to the high growth of commercial and smallholder poultry enterprises. Moreover, in the poultry sector, feed accounts for approximately 60–80% of the total cost (3), with fish and soybean meal used as the main protein sources. However, an increase in the world's population, together with the poultry sector growth, it fails to meet the increasing demand due to feed deficiencies and high costs (4). Thus, it is important to find alternative, cheap protein sources to be used in poultry diets. The main limitations on efficient animal production are due to the high cost and lack of availability of commercial protein sources, which result in these protein sources being less accessible. Moreover, the utilization of meat and bone meal adds to a group of less accessibility protein sources, such as fishmeal and soybean, which means that a huge market for alternative protein sources is there (4). Therefore, future approaches in identifying alternative cheap and readily available protein sources are recommended. Leafy vegetables, such as *Amaranthus spp*., are the cheapest and most readily available source of basic nutrients, such as proteins, vitamins, minerals, and essential amino acids (5). *Amaranthus* is a volunteer crop that grows immediately after the first summer rains. This crop is grown extensively as a leafy vegetable and grains in rural areas, where chickens are mostly reared for human consumption. However, amaranth is also known to have antinutritional factors, such as trypsin inhibitors, phenols, tannins, and saponins (3, 6, 7). The levels of these antinutritional factors can be reduced by utilizing processing methods, such as autoclaving, cooking, popping, and extruding, especially in grains (3, 7, 8). It has also been reported that amaranth vegetables have been used in many countries as a grain, forage, or silage crop for many animals, including cattle, chickens, pigs, and rabbits (9). Calves performed well when given diets containing up to 40% amaranth leaf meal with a comparable feeding value to that of lucerne meal. Lebas (10) reported an acceptable effect on growth when the *Amaranthus* crop was used as a component of the New Zealand white rabbit rations at up to 40% inclusion level, whereas Molina et al. (11) reported that the health status and weight gain or live weight of the rabbits were not affected by the changes in the amaranth inclusion rate up to 32%. The addition of 10% of *Amaranthus cruentus* hydrolysate to the pig feed ratio has been reported to increase the digestibility, the degree of assimilation of nitrogen, and the productivity of weaners (12). However, Longato et al. (13) evaluated the growth performance, blood serum metabolites, oxidative status, and meat quality of broilers fed diets containing 0, 5, and 10% of *Amaranthus*, respectively. The authors reported no differences in the alanine aminotransferase and albumin levels or the meat quality characteristics. However, its potential as a protein source in poultry diets has not been fully explored and, thus, results on its use remain inconclusive. Hence, the objective of this study was to determine the effect of varying inclusion levels of amaranth leaf meal on the performance, blood profiles, and gut organ characteristics of Ross 308 broiler chickens.

## Materials and Methods

### Study Site

This study was conducted at the University of Limpopo Animal Unit (latitude of 27.55°S and longitude of 24.77°E). The ambient temperature at the study site ranges between 20 and 36°C in the summer months (November to January) and between 5 and 25°C in the winter months (May to July). Mean annual rainfall ranges between 446.8 and 468.44 mm. This study was conducted during the winter period.

### Preparation of the House

A total of 200, day-old, male Ross 308 broiler chicks were brought from a local hatchery. The chickens were housed in an open-sided structure and the long axis was situated along an east-west direction for proper ventilation in 1 m^2^ pens constructed using wire mesh. Moreover, the house was a controlled house with temperatures maintained at 30 to 33°C and 23 to 25°C during the starter and grower phases, respectively. Paraformaldehyde was used to disinfect the poultry house 2 weeks before the start of the experiment. Wood shaved sawdust was used as bedding for the chickens. Drinkers and feeders were washed and cleaned with disinfectants daily in the morning before being used. The experimental period lasted for 42 days. Feed and water were provided *ad libitum*.

### Experimental Diets, Design, and Procedures

A total of 200, day-old, Ross 308 broiler chicks with an initial live weight of 42 ± 8 g were received from a local hatchery and randomly allocated to five dietary treatment levels in a complete randomized design, with each treatment group consisting of four replicates and each replicate of 10 chicks. Amaranth leaf meal (ALM) inclusion levels were at 0, 5, 10, 15, and 20%. *Amaranthus cruentus* (L) leaves, which were used in this study, were grown under a controlled field trial in the North-West Province, South Africa. The mean temperatures around the area are above 22°C in summer and below 20°C in winter and lie at a latitude of 25.6200 °S and longitude of 27.9800 °E. The aforementioned variety was grown in September 2019 under dry land conditions, which receives a mean annual rainfall of <250 mm. Amaranth leaves were hand-harvested. Thereafter, harvested leaves were independently dried in a well-ventilated laboratory to obtain a constant weight and milled through a 1-mm sieve into powder, by using a hammer mill, before being analyzed (Table 1) and incorporated into the formulated diets (Table 2).

**Table 1:** Proximate composition (g/100 g), gross energy (kcal/g), and amino acids composition (%) of *Amaranthus cruentus* leaf meal (ACLM).

**Composition (g/100 g)**	**ACLM**
DM	92.65
CP	23.23
CF	17.14
NDF	15.40
ADF	7.14
ADL	1.95
GE	14.50
EE	1.12
Starch	0.38
Ash	21.18
**Amino acids**	
Histidine	0.29
Arginine	0.90
Threonine	0.85
Lysine	1.73
Tyrosine	0.52
Methionine	0.34
Valine	1.51
Leucine	1.55
Serine	0.90
Glycine	0.94
Aspartic acid	2.16
Glutamine	2.94
Alanine	1.27
Proline	0.87
Isoleucine	0.83
Phenylalanine	0.66

*Values are means of duplicate analyzed amaranth leaves samples*.

**Table 2:** Ingredients and calculated analysis of experimental diets.

**Amaranth leaf meal (ALM) inclusion levels (%)**					
**Ingredients**	**0**	**5**	**10**	**15**	**20**
Maize	40	40	40	40	40
Groundnut oil cake	10	18	15	12	10
Soya bean meal	8	8	8	8	8
Fish meal	2	2	2	2	2
Wheat offals	15.7	22.7	20.7	18.7	15.7
Bone meal	2.5	2.5	2.5	2.5	2.5
Limestone	0.5	0.5	0.5	0.5	0.5
Salt (NaCl)	0,5	0.5	0.5	0.5	0.5
Dl-Methionine	0.15	0.15	0.15	0.15	0.15
L-Lysine	0.15	0.15	0.15	0.15	0.15
Vit/Min Premix^†^	0.5	0.5	0.5	0.5	0.5
ALM	0	5	10	15	20
Total	100	100	100	100	100
**Calculated analysis**					
Crude protein (%)	20.00	20.00	20.00	20.00	20.00
Crude fiber (%)	4.52	4.57	4.71	4.70	4.73
Ether extract (%)	7.21	6.41	6.40	6.71	6.51
GE (kcal/100 g)	462.4	462.3	462.1	461.5	461.7
**Analyzed composition**					
Crude protein (%)	19.44	19.50	19.51	19.48	20.01
Crude fiber (%)	5.03	6.22	6.35	6.43	6.37
Ether extract (%)	7.61	7.65	7.80	7.81	7.79
GE (kcal/100 g)	453.7	452.80	452.70	451.60	451.00

† *The ingredients contained in the vitamin–mineral premix were as follows (per kg of diet): vitamin A 12,000 IU, vitamin D3 3,500 IU, vitamin E 30.0 mg, vitamin K3 2.0 mg, thiamine 2 mg, riboflavin 6 mg, pyridoxine 5 mg, vitamin B12 0.02 mg, niacin 50 mg, pantothenate 12 mg, biotin 0.01 mg, folic acid 2 mg, Fe 60 mg, Zn 60 mg, Mn 80 mg, Cu 8 mg, Se 0.1 mg, Mo 1 mg, Co 0.3 mg, I 1 mg*.

### Data Collection

#### Growth Performance

The live weight of each chicken was determined at the start of the experiment; thereafter, weekly weights were taken. The daily feed intake (FI) was determined by subtracting the weight of feed leftover from the total weight of the feed that was given to chickens daily and the difference was divided by the total number of chickens in each replicate for 6 weeks. The feed conversion ratio was then calculated using the following formulae:


$$\begin{array}{l} \text{Feed Conversion Ratio }\left(\text{FCR}\right)=\frac{\text{Feed intake }\left(\text{g}\right)}{\text{Bodyweight }\left(\text{g}\right)} \end{array}$$

#### Apparent Nutrient Digestibility

Apparent nutrient digestibility measurements were carried out when the chickens were between the ages of 37 and 42 days; excreta were collected in trays beneath each metabolic cage following 48 h, dried at 70°C in an oven for 48 h, and then weighed to determine nutrients digestibility. Proximate analysis for moisture, ash, crude protein (N × 6.25), fat, and crude fiber was determined according to standardized methods of the Association of Official Analytical Chemists (AOAC) (2012). Amaranth leaves flour samples were oven-dried and weighed, before being ashed in a muffle furnace at 550°C for 6 h. The ash was acid digested by adding 1 ml 55% (v/v) HNO_3_. The gross energy content of the milled samples was determined with adiabatic bomb calorimetry (Gallenkamp, Autobomb, and London, UK). Ether extracted lipid content was estimated using Tecator Soxtec. Amino acid separation and detection were performed *via* the Waters ACQUITY ultra-performance liquid chromatography (UPLC), fitted with a photodiode array (PDA) detector. This required 1 μl of sample/standard solution injected into the mobile phase, which conveyed derivatized amino acids onto the Waters UltraTax C18 Column (2.1 mm × 50 mm × 1.7 μm) maintained at 60°C. Elution of analytes off the column was performed by running a gradient. Analytes eluting off the column were detected by the PDA detector, with individual amino acids coming off the column at unique retention times.

#### Blood Collection

On day 42, blood samples of 60 birds (three birds per pen) per feeding group were collected from chickens. A total of 2.5 ml was placed in an EDTA tube. A blood smear was prepared using a glass slide for each chicken, from a drop of blood, containing an anticoagulant. The smears were stained using May–Grünwald and Giemsa stains (14). The total blood cells counts were determined in an improved Neubauer hemocytometer (Merck Sigma-Aldrich).

#### Gut Organ Characteristics

On days 21 and 42, three chickens per pen were slaughtered using the cervical dislocation method, following the recommendations of the University of Limpopo and the University of South Africa's ethical guidelines. The birds were immersed in hot water to remove the feathers and then cleaned and dissected to harvest the internal organs. Gut organ lengths were measured using a measuring tape, while gut organ weights were measured using an electronic weighing scale and gut organ pH was measured using the digital pH meter (Crison, Basic 20 pH Meter).

#### Statistical Analysis

The statistical analysis was performed using the general linear model (GLM) procedure of SAS (15). Where there were significant differences (*P* < 0.05), the treatment means were separated using the Duncan's test at a 5% level of probability. Furthermore, collected data were evaluated for linear and quadratic effects using polynomial contrasts.

The quadratic models were fitted to the experimental data by using the procedure of SPSS (16). The response in optimum measured parameters of the Ross 308 broiler chickens, due to the inclusion of amaranth leaf meal, was modeled using the following quadratic equation:


$$\begin{array}{l} Y=a+b1x+b2x2 \end{array}$$

Where y = optimum, a = intercept; b = coefficients of the quadratic equation; x = *Amaranthus* meal inclusion level; and -b1/2b1 = x value for optimum response. The quadratic equation was the preferred model, as it gives the optimum fit.

## Results

### Performance and Blood Profiles Results of Broiler Chickens

The results of the effect of ALM inclusion levels on feed intake, body weight (BW), and the feed conversion ratio of Ross 308 broiler chickens are given in Table 3. ALM inclusion levels had no effect (*p* > 0.05) on feed intake, body weight, or feed conversion ratios of Ross 308 broiler chickens between 1–21 and 22–42 days, respectively. However, even though there was no significant difference observed between treatment and feed intake or body weight and feed conversion ratios, there was a positive quadratic (*p* = 0.050, 0.045, and 0.014, respectively) influence on FI at the age of 21 days and FI and BW at the age of 42 days.

**Table 3:** Effect of amaranth leaf meal inclusion on feed intake (FI) (g/bird), weight gain (gain, g/bird), and feed conversion ratio (FCR) (g:g, FI:BWG) of Ross 308 broiler chickens.

	**1–21 days**			**22–42 days**		
**ALM%**	**FI**	**BW**	**FCR**	**FI**	**BW**	**FCR**
0	135.63	129.54	1.08	946.18	1763.2	1.86
5	142.47	123.50	1.15	989.51	1834.9	1.85
10	139.73	138.81	1.01	995.24	1830.6	1.83
15	139.23	138.52	1.01	984.26	1794.3	1.82
20	129.10	123.42	1.05	884.58	1712.5	1.94
SEM	5.142	7.843	0.075	41.143	76.008	0.181
***P*** **-Value**						
Treatment	0.101	0.682	0.695	0.285	0.771	0.485
Linear	0.311	0.927	0.345	0.460	0.457	0.466
Quadratic	0.050	0.648	0.672	0.045	0.014	0.234

*Diets: ALM 0% = a diet having no amaranth leaf meal inclusion, ALM 5% = a diet having 5 g/kg of amaranth leaf meal inclusion, ALM 10% = a diet having 10 g/kg of amaranth leaf meal inclusion. ALM 15% = a diet having 15 g/kg of amaranth leaf meal inclusion. ALM 20% = a diet having 20 g/kg of amaranth leaf meal inclusion. Values are means of three replicates analyzed. SEM, standard error of the mean*.

Blood profiles of Ross 308 broiler chickens that were fed with diets with ALM inclusion levels are shown in Table 4. Ross 308 broiler chickens, which were fed with a 5% ALM inclusion level, had higher (*p* < 0.05) white blood cells (WBCs) and lymphocyte counts than those fed with diets containing 0, 10, 15, and 20% levels. Ross 308 broiler chickens on diets with a 15% ALM inclusion level had higher (*p* < 0.05) monocytes and basophils than those on diets with 0, 5, 10, and 20% levels. Chickens that were provided diets with a 20% ALM inclusion level had higher (*p* < 0.05) heterophils than chickens fed with 5, 10, and 15% diets. There was no linear or quadratic effect observed in any of the blood profiles of Ross 308 broiler chickens with increasing levels of ALM in their diets.

**Table 4:** Effect of amaranth inclusion leaf meal on blood profiles of Ross 308 broiler chickens.

	**WBC**	**Hetero** **phils**	**Lympho** **cytes**	**Monocytes**	**Eosino** **phils**	**Basophils**
**ALM%**						
0	24.38^b^	51.25^ab^	37.75^cd^	2.50^bc^	0.00^b^	5.75^c^
5	26.28^a^	36.75^c^	61.50^a^	1.75^c^	1.00^a^	2.00^d^
10	17.25^d^	25.50^d^	44.50^bc^	3.25^b^	0.00^b^	7.50^b^
15	20.75^c^	42.25^bc^	48.00^b^	6.00^a^	1.00^a^	9.25^a^
20	17.48^d^	58.75^a^	33.00^d^	1.75^c^	0.00^b^	6.75^bc^
SEM	0.419	3.430	2.877	0.296	0.00	0.433
***P*** **-value**						
Treatment	<0.000	<0.000	<0.000	<0.000	<0.000	<0.000
Linear	0.140	0.683	0.584	0.689	0.528	0.345
Quadratic	0.425	0.062	0.404	0.749	0.762	0.702

*a, b, c, d Means in the same row not sharing a common superscript are different (p < 0.05). Diets: ALM 0% = a diet having no ALM inclusion, ALM 5% = a diet having 5 g/kg of ALM inclusion, ALM 10% = a diet having 10 g/kg of ALM inclusion. ALM 15% = a diet having 15 g/kg of ALM inclusion. ALM 20% = a diet having 20 g/kg of ALM inclusion. Values are means of three replicates analyzed. SEM, standard error of the mean*.

The findings of the influence of ALM on nutrient digestibility of Ross 308 broiler chickens are shown in Table 5. ALM inclusion levels had no effect (*p* < 0.05) on dry matter (DM) or gross energy (GE) digestibility of Ross 308 broiler chickens. Ross 308 broiler chickens, which were fed with diets having a 5% ALM inclusion level, had higher (*p* < 0.05) crude fiber (CF), crude protein (CP), and ash digestibility values than those fed with diets containing 0, 10, 15, and 20% ALM inclusion levels. Ross 308 broiler chickens that were fed with diets having a 10% ALM inclusion level had higher (*p* < 0.05) ether extracts (EEs) digestibility values than those fed with diets containing 0, 5, 15, and 20% ALM inclusion levels. Interestingly, there was no linear or quadratic effect observed in any nutrient digestibility of Ross 308 broiler chickens with increasing levels of ALM in their diets.

**Table 5:** Effect of amaranth leaf meal (ALM) inclusion on nutrient digestibility (%) of Ross 308 broiler chickens.

		**Nutrient digestibility**				
**ALM%**	**DM (%)**	**CP (%)**	**GE (MJ/kg)**	**CF (%)**	**EE (%)**	**Ash (%)**
0	93.91	73.43^c^	15.74	62.78^c^	36.08^c^	57.86^c^
5	94.54	79.68^a^	16.67	65.09^a^	38.09^b^	60.32^a^
10	94.50	74.47^b^	16.53	64.35^b^	38.57^a^	58.42^b^
15	93.86	68.97^d^	15.93	61.50^e^	21.37^d^	56.45^d^
20	93.86	62.54^e^	15.71	61.71^d^	12.67^e^	55.42^e^
SEM	0.012	0.012	0.012	0.015	0.012	0.011
***P*** **-value**
Treatment	0.061	<0.000	0.059	<0.000	<0.000	<0.000
Linear	0.565	0.105	0.649	0.314	0.063	0.157
Quadratic	0.383	0.289	0.258	0.646	0.065	0.209

*a, b, c, d, e Means in the same row not sharing a common superscript are different (p < 0.05). Diets: ALM 0% = a diet having no amaranth leaf meal inclusion, ALM 5% = a diet having 5 g/kg of amaranth leaf meal inclusion, ALM 10% = a diet having 10 g/kg of amaranth leaf meal inclusion. ALM 15% = a diet having 15 g/kg of amaranth leaf meal inclusion. ALM 20% = a diet having 20 g/kg of amaranth leaf meal inclusion. Values are means of three replicates analyzed. SEM, standard error of the mean*.

The results of the effects of ALM inclusion levels on essential amino acids digestibility are shown in Table 6. ALM inclusion levels had an effect (*p* < 0.05) on the essential amino acid digestibility of Ross 308 broiler chickens. Thus, birds fed with diets having 5 and 10% ALM inclusion levels had higher (*p* < 0.05) Arg, His, Leu, Lys, Met, Phen, Thr, and Val digestibility than those on 0, 15, and 20% ALM inclusion levels. There was a positive quadratic (*p* = 0.041, 0.008, and 0.034) effect on the Phen, Thr, and Val digestibility of Ross 308 broiler chickens with increasing levels of ALM in their diets. Moreover, the Phen, Thr, and Val digestibility was optimized (0.932, 0.35, and 0.646%) at amaranth inclusion levels of 8.75, 19.74, and 6.667% (Table 7).

**Table 6:** Effect of amaranth leaf meal inclusion on essential amino acids digestibility (%) of Ross 308 broiler chickens.

**Essential amino acid digestibility %**									
**ALM%**	**Arg**	**His**	**Ile**	**Leu**	**Lys**	**Met**	**Phen**	**Thr**	**Val**
0	0.72^c^	0.62^d^	0.28^c^	0.33^b^	0.40^d^	0.61^b^	0.46^c^	0.28^c^	0.23^c^
5	0.83^a^	0.81^a^	0.44^a^	0.43^a^	0.66^a^	0.78^a^	0.62^a^	0.44^a^	0.44^a^
10	0.81^ab^	0.78^a^	0.39^b^	0.41^a^	0.61^ab^	0.77^a^	0.62^a^	0.42^a^	0.42^a^
15	0.78^b^	0.73^b^	0.36^b^	0.40^a^	0.57^b^	0.61^b^	0.50^b^	0.33^b^	0.32^b^
20	0.74^c^	0.69^c^	0.19^d^	0.32^b^	0.50^c^	0.47^c^	0.36^d^	0.10^d^	0.13^d^
SEM	0.012	0.011	0.012	0.012	0.012	0.012	0.012	0.012	0.012
***P*** **-value**									
Treatment	<0.000	<0.000	<0.000	<0.000	<0.000	<0.000	<0.000	<0.000	<0.000
Linear	0.956	0.839	0.484	0.798	0.783	0.337	0.440	0.342	0.518
Quadratic	0.193	0.253	0.069	0.089	0.236	0.064	0.041	0.008	0.034

*a, b, c, d Means in the same row not sharing a common superscript are different (p < 0.05). Diets: ALM 0% = a diet having no amaranth leaf meal inclusion, ALM 5% = a diet having 5 g/kg of amaranth leaf meal inclusion, ALM 10% = a diet having 10 g/kg of amaranth leaf meal inclusion. ALM 15% = a diet having 15 g/kg of amaranth leaf meal inclusion. ALM 20% = a diet having 20 g/kg of amaranth leaf meal inclusion. Values are means of three replicates analyzed. SEM, standard error of the mean*.

**Table 7:** Relationships between amaranth leaf meal inclusion and essential amino acids digestibility.

**Parameter**	**Formula**	***r*** **^2^**	**X-value**	**Optimal value**	***P*-value**
Phen	Y = 0.473+ 0.035x+-0.002x^2^	0.959	8.75	0.93	0.041
Thr	Y = 0.105+0.354x+0.0009x^2^	0.972	19.74	0.35	0.008
Val	Y = 0.246+0.04x+-0.003x^2^	0.966	6.67	0.65	0.034

*r^2^ = coefficient of determination*.

The nonessential amino acids digestibility of Ross 308 broiler chickens, which were fed varying ALM inclusion levels, are shown in Table 8. ALM inclusion levels had an effect (*p* < 0.05) on the nonessential amino acids digestibility of Ross 308 broiler chickens. Ross 308 broiler chickens, which were fed with diets having a 5% ALM inclusion level, had higher (*p* < 0.05) Ala, Glu, Gly, Pro, Ser, and Tyr digestibility than those on 0, 10, 15, and 20% ALM inclusion levels. Ross 308 broiler chickens, which were given diets containing a 20% ALM inclusion level, had higher (*p* < 0.05) Asp digestibility than those on 0, 5, 10, and 15% ALM inclusion levels. There was a positive linear (*p* = 0.004) and quadratic (*p* = 0.045) effect on Asp digestibility of Ross 308 broilers with increasing levels of ALM in their diet.

**Table 8:** Effect of amaranth leaf meal inclusion level on nonessential amino acids digestibility (%) of Ross 308 broiler chickens.

**Nonessential amino acid digestibility (%)**							
**ALM%**	**Ala**	**Asp**	**Glu**	**Gly**	**Pro**	**Ser**	**Tyr**
0	0.43^c^	0.30^c^	0.57^d^	0.25^e^	0.17^c^	0.27^c^	0.23^d^
5	0.74^a^	0.31^c^	0.70^a^	0.66^a^	0.41^a^	0.52^a^	0.67^a^
10	0.62^b^	0.45^c^	0.61^c^	0.29^d^	0.33^b^	0.27^c^	0.65^a^
15	0.61^b^	0.51^b^	0.67^ab^	0.52^c^	0.31^b^	0.43^b^	0.47^b^
20	0.60^b^	0.58^a^	0.66^b^	0.56^b^	0.20^c^	0.50^b^	0.32^c^
SEM	0.012	0.012	0.012	0.012	0.012	0.012	0.017
***P*** **-value**							
Treatment	<0.000	<0.000	<0.000	<0.000	<0.000	<0.000	<0.000
Linear	0.624	0.004	0.437	0.474	0.918	0.412	0.979
Quadratic	0.501	0.045	0.676	0.807	0.240	0.765	0.159

*a, b, c, d Means in the same row not sharing a common superscript are different (p < 0.05). Diets: ALM 0% = a diet having no amaranth leaf meal inclusion, ALM 5% = a diet having 5 g/kg of amaranth leaf meal inclusion, ALM 10% = a diet having 10 g/kg of amaranth leaf meal inclusion. ALM 15% = a diet having 15 g/kg of amaranth leaf meal inclusion. ALM 20% = a diet having 20 g/kg of amaranth leaf meal inclusion. Values are means of three replicates analyzed. SEM, standard error of the mean*.

### Gut Organ Characteristics

The effects of ALM inclusion levels on gut organ weights of Ross 308 broiler chickens are shown in Table 9. ALM inclusion levels had no effect (*p* > 0.05) on gastrointestinal tract (GIT), crop, progizzard, gizzard, liver, spleen, small intestine, or ceca weights of Ross 308 broiler chickens aged 21 and 42 days. ALM inclusion levels had an effect (*p* < 0.05) on large intestine weights of Ross 308 broiler chickens aged 21 days. Ross 308 broiler chickens, which were fed with diets having 5 and 10% ALM inclusion levels, had heavier (*p* < 0.05) large intestines than those on 15 and 20% ALM inclusion levels. Moreover, there was no linear (*p* > 0.05) or quadratic (*p* > 0.05) influences on any of the gut organ weights of Ross 308 broiler chickens measured with increasing levels of ALM in the diet.

**Table 9:** Effect of amaranth leaf meal inclusion on gut organ weights (g) of Ross 308 broiler chickens.

**Parameters**									
**ALM%**	**GIT**	**Crop**	**ProGiz**	**Gizz**	**Liver**	**Spleen**	**SI**	**Caeca**	**LI**
**21 days**
0	68.13	3.83	3.85	16.25	12.35	0.38	36.25	3.75	0.95^b^
5	76.60	4.90	3.98	19.38	13.90	0.53	37.70	4.35	1.53^ab^
10	78.45	5.00	4.40	19.38	14.75	0.50	42.55	5.00	2.25^a^
15	79.70	5.08	4.75	20.58	16.75	0.53	43.15	5.50	0.98^b^
20	74.78	4.80	3.95	18.38	13.13	0.48	37.43	4.15	0.93^b^
SEM	10.101	1.131	0.545	2.215	2.105	0.094	4.666	0.696	0.310
***P*** **-value**									
Treatment	0.933	0.932	0.747	0.712	0.638	0.779	0.759	0.437	0.035
Linear	0.314	0.228	0.499	0.356	0.491	0.379	0.520	0.456	0.794
Quadratic	0.064	0.059	0.374	0.109	0.304	0.200	0.243	0.202	0.339
**42 days**
0	188.74	7.23	8.45	44.50	47.73	2.43	86.39	12.26	3.34
5	212.04	10.63	10.41	50.48	52.79	3.13	112.15	15.36	4.55
10	204.94	11.75	9.59	47.58	52.75	3.04	104.29	15.23	4.40
15	197.85	9.06	9.40	46.20	51.18	2.85	96.73	14.11	4.21
20	190.30	8.45	8.69	45.30	50.95	2.79	94.81	12.84	3.90
SEM	14.424	1.565	0.861	3.922	3.801	0.352	11.935	1.487	0.647
***P*** **-value**									
Treatment	0.760	0.313	0.534	0.835	0.878	0.671	0.622	0.502	0.701
Linear	0.755	0.902	0.863	0.771	0.539	0.677	0.971	0.987	0.676
Quadratic	0.267	0.211	0.334	0.482	0.245	0.298	0.433	0.126	0.198

*a, b Means in the same row not sharing a common superscript are significantly different (p < 0.05). Diets: ALM 0% = a diet having no amaranth leaf meal inclusion, ALM 5% = a diet having 5 g/kg of amaranth leaf meal inclusion, ALM 10% = a diet having 10 g/kg of amaranth leaf meal inclusion. ALM 15% = a diet having 15 g/kg of amaranth leaf meal inclusion. ALM 20% = a diet having 20 g/kg of amaranth leaf meal inclusion. Values are means of three replicates analyzed. SEM, standard error of the mean*.

The gut organ lengths of Ross 308 broiler chickens, which were fed with ALM inclusion in their diets, are shown in Table 10. ALM inclusion levels had no effect (*p* > 0.05) on GIT, small intestine, ceca, or large intestine lengths of Ross 308 broiler chickens aged 21 and 42 days. Furthermore, gut organ lengths of Ross 308 broiler chickens measured showed no linear (*p* > 0.05) or quadratic (*p* > 0.05) influences with increasing levels of ALM in the diet. The effects of ALM inclusion levels on gut organ digesta pH of Ross 308 broiler chickens are shown in Table 11. ALM inclusion levels did not affect (*p* > 0.05) digesta pH of the crop, progizzard, gizzard, small intestine, ceca, or large intestine of Ross 308 broiler chickens aged 21 and 42 days. ALM inclusion levels in the diet showed no linear or quadratic influences on gut organ digesta pH of Ross 308 broiler chickens.

**Table 10:** Effect of amaranth leaf meal inclusion on gut organ lengths (cm) of Ross 308 broiler chickens.

	**Parameters**			
**ALM%**	**GIT**	**SI**	**Caeca**	**LI**
**1–21 days**
0	137.38	11.81	119.66	6.13
5	161.13	13.60	137.35	8.28
10	160.00	13.39	137.13	7.31
15	156.13	13.06	134.31	6.93
20	155.50	12.80	134.13	6.63
SEM	5.617	0.597	4.728	0.492
***P*** **-value**				
Treatment	0.056	0.294	0.095	0.071
Linear	0.376	0.592	0.329	0.913
Quadratic	0.224	0.241	0.202	0.488
**22–42 days**
0	247.13	198.75	19.76	11.44
5	261.75	208.38	20.88	12.88
10	254.13	208.63	22.00	12.13
15	249.13	207.38	20.75	12.00
20	248.00	198.75	20.56	11.38
SEM	8.142	10.978	1.487	0.955
***P*** **-value**				
Treatment	0.697	0.915	0.599	0.733
Linear	0.644	0.961	0.637	0.673
Quadratic	0.516	0.059	0.229	0.343

*Diets: ALM 0% = a diet having no amaranth leaf meal inclusion, ALM 5% = a diet having 5 g/kg of amaranth leaf meal inclusion, ALM 10% = a diet having 10 g/kg of amaranth leaf meal inclusion. ALM 15% = a diet having 15 g/kg of amaranth leaf meal inclusion. ALM 20% = a diet having 20 g/kg of amaranth leaf meal inclusion. Values are means of three replicates analyzed. SEM, standard error of the mean*.

**Table 11:** Effect of amaranth leaf meal inclusion on gut organ digesta pH of Ross 308 broiler chickens.

	**Parameters**					
**ALM%**	**Crop**	**ProGizz**	**Gizz**	**SI**	**Caeca**	**LI**
**1–21 days**
0	4.39	3.84	2.87	5.24	6.14	5.89
5	4.65	3.78	3.48	5.64	6.29	6.15
10	4.60	3.55	3.12	5.28	6.25	5.96
15	4.60	3.76	3.38	5.53	6.25	5.97
20	5.03	3.98	4.78	5.67	6.34	6.28
SEM	0.154	0.189	0.492	0.197	0.106	0.123
***P*** **-value**						
Treatment	0.113	0.620	0.112	0.436	0.758	0.190
Linear	0.078	0.668	0.108	0.295	0.126	0.294
Quadratic	0.252	0.193	0.211	0.648	0.382	0.588
**22–42 days**
0	4.52	4.09	4.19	3.69	3.28	5.75
5	5.24	4.55	4.48	4.33	3.88	6.03
10	4.75	4.28	4.22	3.73	3.60	5.85
15	5.25	4.55	4.41	3.95	3.66	6.03
20	5.80	4.84	4.62	4.64	4.55	6.16
SEM	0.297	0.359	0.224	0.472	0.428	0.173
***P*** **-value**						
Treatment	0.352	0.542	0.201	0.631	0.531	0.119
Linear	0.092	0.085	0.195	0.296	0.125	0.107
Quadratic	0.303	0.309	0.474	0.563	0.334	0.363

*Diets: ALM 0% = a diet having no amaranth leaf meal inclusion, ALM 5% = a diet having 5 g/kg of amaranth leaf meal inclusion, ALM 10% = a diet having 10 g/kg of amaranth leaf meal inclusion. ALM 15% = a diet having 15 g/kg of amaranth leaf meal inclusion. ALM 20% = a diet having 20 g/kg of amaranth leaf meal inclusion. Values are means of three replicates analyzed. SEM, standard error of the mean*.

## Discussion

Amaranth crop is known as a vegetable protein and grown in most tropical regions of the world. In this study, varying ALM inclusion levels had no effect on feed intake, body weight, or feed conversion ratios of Ross 308 broiler chickens aged between 1–21 and 22–42 days, respectively. The results of this study are in disagreement with those of Fasuyi (17) who reported that ALM had a positive significant difference in performance characteristics. Although the chickens' body weights, which were obtained in their study, were lower than those found in this study. It is noteworthy that the inclusion of ALM in this study did not affect the performance of experimental chickens. It is possible that the ALM inclusion levels were not too high to adversely affect the performance of chickens. However, the findings of this study are in agreement with the results of Rouckova et al. (18) who did not observe any effect on growth performance and body weight when broiler chickens were fed diets having up to 8% amaranth feed mixture. On the contrary, Pisarikova et al. (19) and Orczewska-Dudek et al. (20) reported that the addition of 8% amaranth to diet mixture reduced the body weight of the broiler chickens, as ALM inclusion levels did not affect dry matter (DM) or gross energy (GE) digestibility of Ross 308 broiler chickens. However, Ross 308 broiler chickens, which were fed with diets having a 5% ALM inclusion level, had higher crude fiber (CF), crude protein (CP), and ash digestibility values than those fed with diets containing 0, 10, 15, and 20% ALM inclusion levels. In contrary to this study, Fasuyi (17), in balance trials on broiler chickens, reported that nutrient digestibility was affected and was favored at an amaranth inclusion level of 10% without any adverse nutritive condition.

Hematological and biochemical blood indices are health properties that can be used to assess the effectiveness of diet supplements (21). According to Adegoke et al. (22), chickens with good health status are likely to show good performance. Moreover, good blood profiles can act as pathological indicators of chickens' responses to toxic substance exposure, as well as organ function. In this study, ALM inclusion affected WBC, heterophils, lymphocytes, monocytes, eosinophils, and basophils of Ross 308 broiler chickens aged 1 to 42 days. Ross 308 broiler chickens, which were fed with diets having a 5% of amaranth leaf inclusion level, had a higher white blood cells (WBCs) count than those on 10, 15, and 20% ALM inclusion levels. However, the results of this study disagree with the results of Fasuyi and Akindahunsi (23). The authors reported that no difference was observed on hematological indices of broiler chickens fed ALM. Ross 308 broiler chickens on a 20% ALM level had higher heterophils than chickens fed with diets having 0, 5, 10, and 15% amaranth inclusion levels. Ross 308 broiler chickens on diets having a 5% ALM inclusion level had higher lymphocytes than those fed with 0, 10, 15, and 20% levels. Ross 308 broiler chickens on diets having a 15% ALM inclusion level had higher monocytes and basophils than those given 0, 5, 10, and 20% ALM levels. Ross 308 broiler chickens, which were fed with diets containing 5 and 15% ALM levels, had higher eosinophils than those on diets having 0, 10, and 20% ALM levels. However, Ari et al. (24) reported depressed packed cell volume, hemoglobin, white blood cells, neutrophils, and lymphocytes of chickens fed diets consisting of amaranth leaves. However, the values reported in their study varied between 95.5 and 99%, which are quite higher than the lymphocytes of this study. The variation might be due to amaranth vegetables being used and since they were grown under different climatic conditions, which, probably, might have affected their nutritional composition. In contrary to the results of this study, Króliczewska et al. (21) reported that no difference was noted in the hemoglobin level and the hematocrit volume in the birds' blood, which were fed amaranth at inclusion levels of 0, 2, 5, and 10%. However, Orczewska-Dudek et al. (20) reported a decrease in plasma glucose levels when chickens were fed with 4 and 7% amaranth inclusion.

Results of this study showed that ALM inclusion levels affected the essential amino acid digestibility of Ross 308 broiler chickens. Ross 308 broiler chickens, which were fed diets having a 5% inclusion level, had shown to have a higher essential and nonessential amino acids digestibility. However, there is no basis for comparison found in the literature. This might be due to the reflection of perhaps low levels of phytochemicals that were present in amaranth leaves used in this study, as ALM inclusion in diets was increasing. According to Manyelo et al. (3), the advantage of the use of amaranth leaves in animal diets, as compared to other conventional cereals, is that amaranth leaves have a relatively high content of proteins, as well as having an appreciable number of amino acids. In this study, the better amino acid digestibility of Ross 308 broiler chickens in diets with a 5% ALM inclusion level may be perhaps attributed to the presence of phytochemical compounds, which might have been present in higher ALM inclusion levels. According to King et al. (25), secondary metabolites such as alkaloids and flavonoids are known to bind with proteins and inhibit their availability to the animal body.

This study reported that ALM inclusion levels did not affect GIT, crop, progizzard, gizzard, liver, spleen, small intestine, or ceca weights of Ross 308 broiler chickens aged 21 days. ALM inclusion levels affected large intestine weights of Ross 308 broiler chickens aged 21 days. Ross 308 broiler chickens fed diets having 5 and 20% ALM inclusion levels had heavier large intestines than those on diets having 0, 5, 10, and 15% levels. ALM inclusion in Ross 308 broiler chicken diets did not affect GIT, crop, progizzard, gizzard, liver, spleen, small intestine, ceca, or large intestine weights of Ross 308 broiler chickens aged 42 days. Moreover, ALM inclusion levels in Ross 308 broiler chicken diets did not affect GIT, small intestine, ceca, or large intestine lengths of Ross 308 broiler chickens aged 21 and 42 days. The result of this study agrees with the results of Fasuyi et al. (26) and Ahaotu et al. (27), whereby the authors reported that no significant difference was observed in the gut organs of chickens fed with amaranth diets. However, the disagreement found in the literature was when broiler chickens were fed with amaranth grains. Pisarikova et al. (19) reported that a positive influence was observed in gut organs of chickens fed with amaranth-based diets.

According to Rodgers et al. (28), chickens that were given high fibrous diets take a long time to adapt to the feed. Chickens do not have rumen to assist in fiber digestion and it is known that their microbial fermentation takes place inside their large intestine. Hence, in this study, it was observed that chickens fed 5 and 10% ALM levels had heavier large intestines at the age of 21 days. However, the same trend was expected to continue as the chickens grow, surprisingly, at the age of 42 days, no significant difference was observed. The reason, therefore, might be that, perhaps, the gut was already developed and each organ played a significant role in nutrient digestion and absorption (28). ALM inclusion levels did not affect the crop, progizzard, gizzard, small intestine, ceca, or large intestine lengths of Ross 308 broiler chickens aged 21 and 42 days. However, this was expected since gut organ weights were not affected. Moreover, it is well known that gut organ functionality depends mostly on the gizzard function. In this study, its weight was not affected and this might be the reason for the gut organs digesta pH not being affected. However, Nkukwana et al. (29) studied the effects of *Moringa oleifera* leaf meal intestinal morphology, digestive organ size, and digesta pH of broiler chickens and reported a significant decrease in pH of gut organ digesta contents. *Moringa oleifera* leaves are reported to have secondary metabolites, which are also present in amaranth leaves and, thus, have the function of supporting the chicken's growth and health (30).

## Conclusion

In conclusion, ALM can be included in broiler chickens without having any adverse effect on the chickens' performance. Moreover, nutrient digestibility distinguished ALM as a potential nutritive feed resource. The inclusion levels of 5, 10, and 15% ALM in broiler diets showed favor in affected parameters. However, future studies are suggested to ascertain the present results.

## Data Availability Statement

The datasets presented in this study can be found in online repositories. The names of the repository/repositories and accession number(s) can be found at: www.unisa.ac.za.

## Ethics Statement

The animal study was reviewed and approved by University of South Africa's (UNISA) Ethics Code for the use of live animals in research, ethics reference number: 2019/CAES_AREC/154 and University of Limpopo (UL) Ethics Committee, reference number: AREC/12/2020: IR.

## Author Contributions

MM: conceptualization. TM: writing—original draft preparation. NS, JN, WW, and MM: review and editing. NS and MM: visualization. All authors have read and agreed to the published version of the manuscript.

## Conflict of Interest

The authors declare that the research was conducted in the absence of any commercial or financial relationships that could be construed as a potential conflict of interest.

## Publisher's Note

All claims expressed in this article are solely those of the authors and do not necessarily represent those of their affiliated organizations, or those of the publisher, the editors and the reviewers. Any product that may be evaluated in this article, or claim that may be made by its manufacturer, is not guaranteed or endorsed by the publisher.
